# Seroconversion to mRNA SARS-CoV-2 vaccines in patients with autoimmune cytopenias and bone marrow failures

**DOI:** 10.1038/s41598-022-11857-7

**Published:** 2022-05-11

**Authors:** Bruno Fattizzo, Marta Bortolotti, Juri Alessandro Giannotta, Dario Consonni, Silvia Cantoni, Wilma Barcellini

**Affiliations:** 1 Hematology Unit, Fondazione IRCCS Ca’ Granda Ospedale Maggiore Policlinico, via Francesco Sforza 35, 20100 Milan, Italy; 2 Department of Oncology and Oncohematology, University of Milan, Milan, Italy; 3 Epidemiology Unit, Fondazione IRCCS Ca’ Granda Ospedale Maggiore Policlinico, Milan, Italy; 4 Dipartimento di Ematologia e Oncologia, Niguarda Cancer Center, ASST Ospedale Niguarda, Milan, Italy

**Keywords:** Autoimmunity, Vaccines, Haematological diseases, Immunological disorders

## Abstract

Data concerning the efficacy of SARS-CoV-2 vaccines in patients with non-oncological hematologic conditions are lacking. These include autoimmune cytopenias (autoimmune hemolytic anemia AIHA, immune thrombocytopenia ITP, and autoimmune neutropenia), and bone marrow failure syndromes (aplastic anemia, low risk myelodysplastic syndromes, and paroxysmal nocturnal hemoglobinuria). These conditions may relapse/reactivate after COVID-19 infection and vaccine. Moreover, they are mainly handled with immunosuppressive drugs that may hamper the response to vaccine. In this study, we prospectively evaluated the rate of seroconversion after mRNA SARS-CoV-2 vaccines in patients with autoimmune cytopenias or bone marrow failure syndrome after 2 ± 1 months from the last vaccine dose. Overall, 149 patients were tested and 135 (91%) seroconverted. The highest proportion of non-responders was observed in Evans syndrome (association of ITP and AIHA) and warm AIHA patients (p = 0.001), in those with lower levels of baseline serum IgG (p = 0.008), and in patients on active therapy with steroids (p = 0.03) who also had lower anti-Spike titers. The latter were inversely related with age, and a positively with lymphocyte counts. Additionally, patients who had received rituximab within 12 months from vaccination showed higher rates of non-response (p = 0.03) as compared to those treated before. Contrarily, cyclosporine alone, complement inhibitors, and bone marrow stimulating agents had no detrimental effect on seroconversion. These data suggest maintaining high vigilance and adherence to preventive/protective measures in this population since a proportion of cases may not respond or exhibit low anti-Spike titers.

## Introduction

There is increasing awareness about the reduced efficacy of SARS-CoV-2 vaccine in patients with hematologic neoplasms. The latter have been shown lower rate of seroconversion both after COVID-19 infection and after mRNA vaccines^1–3^. Most reports regard indolent non-Hodgkin lymphomas (NHL)^4,5^, and patients receiving B-cell depleting therapies, whilst data concerning non-oncological hematologic conditions are lacking^6^. The latter, encompass patients with autoimmune cytopenias (i.e. autoimmune hemolytic anemia AIHA, immune thrombocytopenia ITP, and autoimmune neutropenia AIN) and with bone marrow failure syndromes, including aplastic anemia (AA), low risk myelodysplastic syndromes (LR-MDS), and paroxysmal nocturnal hemoglobinuria (PNH). These conditions are rare and clinically heterogeneous, and several reports of disease exacerbations have been described both after COVID-19 infection and its vaccine^7–11^. More importantly, these diseases are mainly handled with immunosuppressive drugs, including steroids, the anti-CD20 monoclonal antibody rituximab, cytotoxic immunosuppressants such as cyclosporine A, anti-thymocyte globulin (in AA), and complement inhibitors that may hamper the response to vaccine. In this study, we prospectively evaluated the rate of seroconversion after mRNA SARS-CoV-2 vaccines in patients with autoimmune cytopenias or bone marrow failure syndrome undergoing vaccination at a single center in Milan, Italy. We focused on clinical and laboratory risk factors for lower seroconversion that may be used to optimize the timing of vaccination in this patient population.

## Methods

We prospectively evaluated patients with autoimmune cytopenias and bone marrow failure syndromes undergoing SARS-CoV-2 mRNA vaccination from March until October 2021 at two hematologic centers in Milan, Italy. Inclusion criteria were a diagnosis of AIHA, ITP, Evans syndrome (ES, the association of AIHA and ITP, and/or AIN), AA, LR-MDS, or PNH made according to current guidelines^12–15^ and the administration of a SARS-CoV-2 mRNA vaccine in the previous 3 months. The study was conducted in accordance with Helsinki Declaration and patients gave informed consent. Only patients ≥ 18 years of age have been enrolled. The study protocol was approved by the Ethical Committee of Istituto Nazionale per le Malattie Infettive Lazzaro Spallanzani, Rome, Italy with the code HECOVID.

Patients were sampled and tested for anti-Spike and anti-Nucleocapside IgG titer at 2 ± 1 months from the second vaccine dose or after the first vaccine dose if the subject had experienced COVID-19 infection. Seroconversion data were matched with clinical and laboratory variables to assess predictors of non-response.

For statistical analysis, we used Wilcoxon rank-sum and chi-squared test to compare categorical and quantitative variables, respectively. We calculated seroconversion proportions and 95% confidence intervals (CI) using the Agresti-Coull formula. Statistical analyses were performed with Stata 17 (StataCorp. 2021).

### Ethics approval and consent to participate

Ethics approval and consent to participate were obtained for this study.

### Consent for publication

All authors approved present submission.

## Results

**Figure 1:**
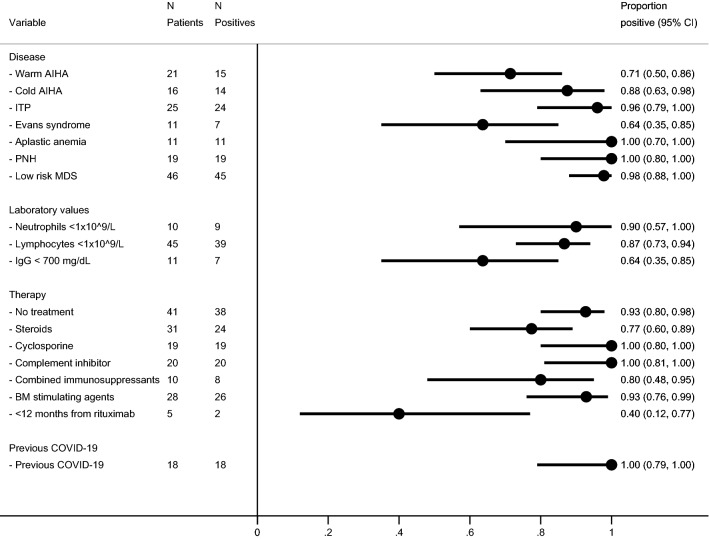
Predictors of seroconversion for anti-Spike IgG in patients with autoimmune cytopenias and bone marrow failure syndromes. AIHA autoimmune hemolytic anemia; MDS myelodysplastic syndromes; ITP immune thrombocytopenia; BM bone marrow.

We enrolled 149 patients (male/female ratio 1.12, median age 73 years, range 17–93) with the following diagnoses: 21 warm type AIHA (wAIHA), 16 cold type AIHA (cAIHA), 25 ITP, 11 ES, 11 AA, 19 PNH, and 46 LR-MDS. Overall, 108 patients were on active treatment, 31 were receiving steroids, 19 cyclosporine A, 20 complement inhibitors (including 3 cAIHA subjects on C1s inhibitor sutimlimab and 17 C5 inhibitors in PNH), 10 cyclosporine combined with steroids, and 28 a bone marrow stimulating agent including the thrombopoietin receptor agonist (TPO-RA) eltrombopag (in ITP) and recombinant erythropoietin (in LR-MDS). The remaining patients were out of therapy: 21 autoimmune cytopenias in remission, and 20 LR-MDS on clinical follow up. Patients received either BNT162b2 (Pfizer-BioNTech, 45%) or mRNA-1273 (Moderna, 55%) vaccine, and 135 (91%) mounted an IgG anti-Spike titer > 0.8 U/mL (the cut-off of our laboratory). As shown in Table 1 and Fig. 1, the highest proportion of non responders was observed in ES and wAIHA patients (36% and 29%, respectively) as compared to < 15% for other diseases (p = 0.001). Non responders displayed lower levels of baseline serum IgG (p = 0.008), whilst neutrophil and lymphocyte counts had no effect. Treatment status had a significant impact (p = 0.03), with a lower rate of seroconversion in patients on active therapy with steroids alone (77%). The detrimental effect of steroids on seroconversion was not clearly related with the dose, as we observed the same rate of non response in patients receiving > or < 20 mg/day of prednisone (25 and 20%, respectively). A similar lower frequency of seroconversion was observed in patients receiving cyclosporine associated with steroids (80%), whilst cyclosporine single agent and complement inhibitors had no effect. No patients were receiving rituximab during vaccination, however 38 had received the drug with a median time from rituximab to vaccination of 24 (1–156) months. Patients who had received rituximab had a higher prevalence of non-response 20% versus 9% compared to rituximab naïve cases (p = 0.04) and those who seroconverted had a longer time from rituximab to vaccination (p = 0.06). Once categorized, patients who had received rituximab within 12 months from vaccination showed higher rates of non response (38% versus 7%, p = 0.03) as compared to those treated before (Fig. 1). Finally, BM stimulating agents had no impact on seroconversion.

**Table 1:** Clinical and laboratory features of enrolled patients divided according to seroconversion status for anti-Spike antibodies.

	All patients N = 149	Seroconverted N = 135	Not seroconverted N = 14
Age, years	73 (17–93)	73 (17–93)	75 (50–86)
Males/females	79/70	73/62	6/8
**Disease**			
Warm AIHA, N (%)	21	15 (71)	6 (29)*
Cold AIHA, N (%)	16	14 (88)	2 (12)
ITP, N (%)	25	24 (96)	1 (4)
Evans syndrome, N (%)	11	7 (64)	4 (36)*
AA, N (%)	11	11 (100)	0 (0)
PNH, N (%)	19	19 (100)	0 (0)
LR-MDS, N (%)	46	45 (98)	1 (2)
**Laboratory values**			
Neutrophils ×10^9^/L	3.1 (0.07–16)	3.07 (0.07–16)	3.6 (0.2–14)
Lymphocytes ×10^9^/L	1.53 (0.37–5.8)	1.5 (0.37–5.8)	1.3 (0.51–5.2)
Total IgG, mg/dL	951 (207–2128)	998 (207–2128)	659 (256–1011)**
**Therapy**			
No treatment, N (%)	41	38 (93)	3 (7)
Steroids, N (%)	31	24 (77)	7 (23)***
Dose, mg/day	7.5 (3–62.5)	7.5 (2.5–37.5)	10 (5–62.5)
Cyclosporine, N (%)	19	19 (100)	0 (0)
Complement inhibitor, N (%)	20	20 (100)	0 (0)
Combined immunosuppressants, N (%)	10	8 (80)	2 (20)***
BM stimulating agents, N (%)	28	26 (93)	2 (7)
< 12 months from rituximab	5	2 (40)	3 (60)***
Time from rituximab, months	24 (1–156)	26 (6–156)	21 (1–72)
Previous COVID-19, N(%)	18	18 (100)	0 (0)*

*p ≤ 0.001; **p = 0.005; ***p < 0.05.

**Figure 2:**
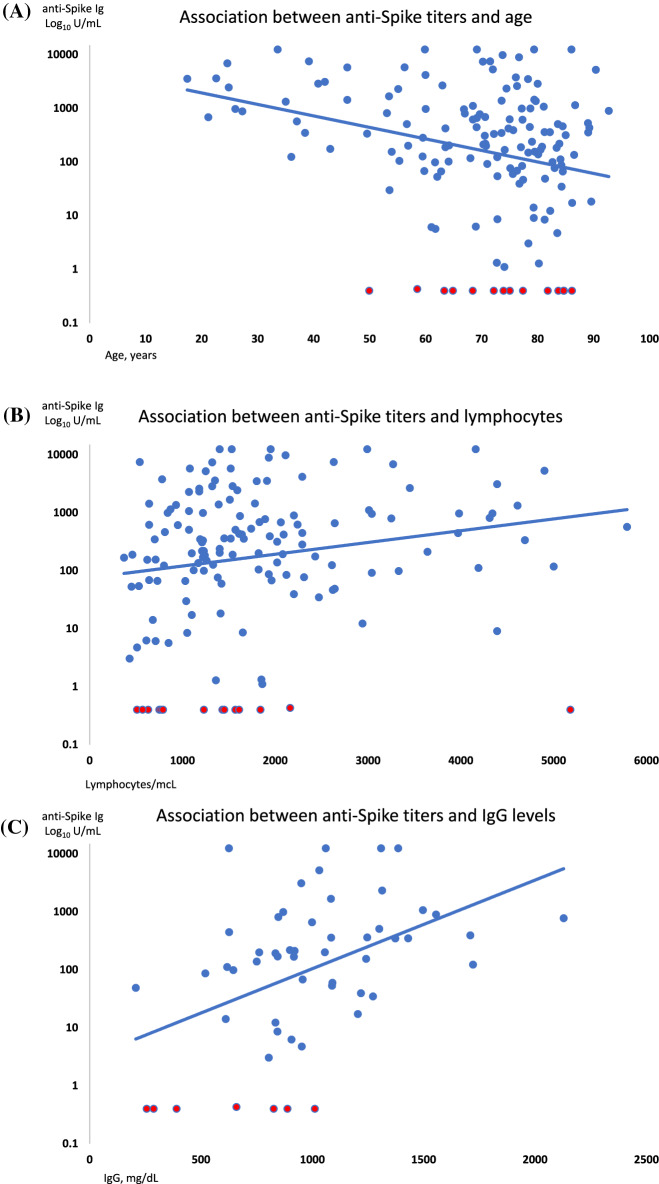
Association among anti-Spike IgG titers and age (**A**), peripheral lymphocyte counts (**B**), and total IgG levels (**C**).

**Figure 3:**
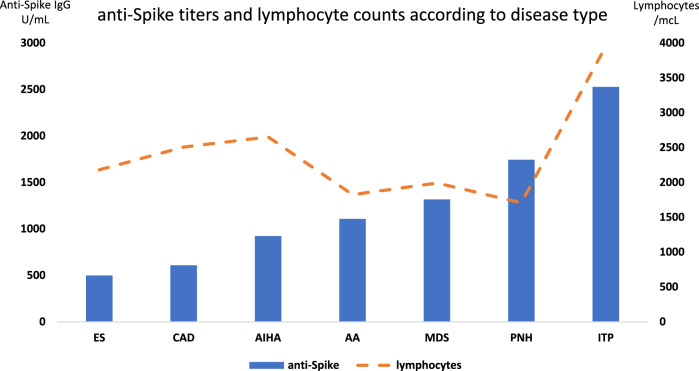
Anti-Spike titers and lymphocyte counts divided according to the various diseases. ES Evans syndrome; CAD cold agglutinin disease; AIHA autoimmune hemolytic anemia; AA aplastic anemia; MDS myelodysplastic syndromes; PNH paroxysmal nocturnal hemoglobinuria; ITP immune thrombocytopenia.

Regarding anti-Spike IgG titers (median 283, 1–40,000 U/mL), a great heterogeneity was noted among responding patients. In particular, an inverse correlation was observed with age (r = − 0.28, p = 0.009), and a positive one with lymphocyte counts (r = 0.47, p = 0.0003) (Fig. 2). Moreover, IgG titers distributed differently across disease types and therapies (Fig. 3): patients with AIHA, ES, and AA had IgG anti-Spike titers below the median of the entire cohort (93, 1.1–12,500 U/mL versus 356, 1–40,000 U/mL, p = 0.04), and those receiving steroids alone or combined to CyA displayed significant lower anti-Spike IgG titers (median 73, 1.1–12,500 U/mL for steroids, 113, 3–12,500 U/mL for cyclosporine plus steroids) as compared to the others (356, 3–40,000 U/mL, p = 0.007), irrespective of steroid dose.

As expected, previous COVID19 infection was associated with a high frequency of anti-Spike seroconversion (100% of cases) and higher median anti-Spike titers (6346 U/mL, 76–40,000 versus 283 U/mL, 1.1–9836, p < 0.001). Anti-Nucleocapside titers were positive in all these cases.

## Discussion

Here we show for the first time that SARS-CoV-2 double-vaccinated patients with autoimmune cytopenias and bone marrow failure syndromes display a frequency of seroconversion (> 90%), nearly comparable to that of the general healthy population^16^. This is in line with recent reports dealing with patients with autoimmune rheumatic diseases that showed seroconversion rates exceeding 80% after 2 doses of anti-SARS-CoV-2 vaccine^17–19^.

From a quantitative perspective, seroconversion was predicted by diseases type, with lower rates of response in subjects with autoantibody mediated diseases (i.e. ES and wAIHA) and maximal response in those with bone marrow failure syndromes (i.e. AA, PNH, and LR-MDS) where autoimmunity is reckoned to be more cellular-mediated. This tendency is also mirrored by the favorable association of seroconversion with higher polyclonal IgG levels pre-vaccine. The latter are a good surrogate of the fitness of the humoral system and are therefore a good predictor of seroconversion possibly useful in clinical practice. On the other hand, therapy had also an effect on seroconversion with patients receiving steroids at the time of vaccine showing lower response rates. Interestingly, the detrimental effect of steroids on seroconversion and anti-Spike titers seemed independent from the dose. This is somewhat unexpected since doses < 20 mg/day of prednisone are usually considered safe and have been hypothesized to have negligible effect on seroconversion by recent guidelines^17–19^. Additionally, impairment of humoral response by B-cell depleting treatment with rituximab within the last 12 months was associated with a worse response, whilst T-cell targeting agent cyclosporine had no effect on seroconversion. Finally, complement inhibitors used in PNH and CAD had also no effect on seroconversion, and this is important given the long-term nature of such therapies. On the whole, our experience is similar to that of rheumatic diseases, where treatment with steroids, rituximab, and mycophenolate mofetil significantly reduced the rate of seroconversion^18,19^. Interestingly, Boekel et al.^17^ observed that repeated exposure to SARS-CoV-2 via infection or vaccination might abrogate the impairment of immune response in autoimmune patients treated with immunosuppressants. Other strategies to improve seroconversion may be the choice of an alternative treatment if available (i.e. TPO-RA versus rituximab in ITP), vaccinating before B-cell depleting drugs, and allowing enough time for immune reconstitution in patients treated with B-cell depleting agents, as already suggested for lymphoproliferative disorders^20,21^. In this regard, Tanguay et al. showed that rates of seroconversion raised to 88% if lymphoma patients had received B-cell depletion > 2 years prior to vaccine (versus 5% in those treated within 1 year before vaccine)^20^.

“Qualitative” response to SARS-CoV-2 vaccine seems more heterogeneous, with some patients exhibiting anti-Spike titers > 10,000 U/mL and other close to the lower cut off of positivity. Lower titers were associated with older age, with autoantibody mediated diseases (ES and wAIHA), with lower lymphocyte counts pre-vaccine, and with ongoing steroid treatment. Although there is great uncertainness regarding the clinical significance of anti-Spike titers to be considered “protective”, it may be hypothesized that lower titers may predict a weaker immune response to SARS-CoV-2 infection. Ferri et al., by studying 478 unselected patients with autoimmune systemic diseases observed significantly lower anti-Spike neutralizing antibodies levels compared to controls (286 (53–1203) versus 825 (451–1542) U/mL) and suggested that these subjects may be named as “suboptimal responders” that should be prioritized for a booster-dose of vaccine, whilst those not responding at all may be administered a different type of vaccine^18^.

Our study carries several limitations mainly regarding the limited number of subjects and the heterogeneity of the diseases included. The latter do however point at the need of tailored approaches in the choice of treatment type and timing to/from vaccine in each condition, rather than a “one size fits all”. Additionally, we did not test for T-cell response to SARS-CoV-2 vaccine, that has been reckoned to lead immune competence in patients undergoing B-cell depletion. In a recent study including patients with autoimmune rheumatic and glomerular diseases, 82.6% of double vaccinated subjects developed a T-cell response that was diminished in those receiving tacrolimus^19^. Thus, it may be hypothesized that our patients treated with CyA, although seroconverted, might have decreased T-cell response to SARS-CoV-2 vaccine, suggesting not to lower preventive measures even in this setting.

In conclusion, patients with autoimmune cytopenias and bone marrow failure syndromes showed high frequency of seroconversion after SARS-CoV2 vaccine. However, high vigilance and adherence to preventive/protective measures (use of personal protective equipment and social distancing) are pivotal since a proportion of cases may not respond or exhibit low anti-Spike titers. These cases are mainly those with impaired humoral immunity as shown by reduced polyclonal IgG or lymphocytes pre-vaccine, and those on active steroid therapy or who received rituximab within the last 12 months.

## Data Availability

All data are available within the manuscript and further may be available upon reasonable request to the corresponding author.
